# NFAT1 C-Terminal Domains Are Necessary but Not Sufficient for Inducing Cell Death

**DOI:** 10.1371/journal.pone.0047868

**Published:** 2012-10-26

**Authors:** Douglas V. Faget, Pedro I. Lucena, Bruno K. Robbs, João P. B. Viola

**Affiliations:** Program of Cellular Biology, Brazilian National Cancer Institute (INCA), Rio de Janeiro, Brazil; Faculdade de Medicina, Universidade de São Paulo, Brazil

## Abstract

The proteins belonging to the nuclear factor of activated T cells (NFAT) family of transcription factors are expressed in several cell types and regulate genes involved in differentiation, cell cycle and apoptosis. NFAT proteins share two conserved domains, the NFAT-homology region (NHR) and a DNA-binding domain (DBD). The N- and C-termini display two transactivation domains (TAD-N and TAD-C) that have low sequence similarity. Due to the high sequence conservation in the NHR and DBD, NFAT members have some overlapping roles in gene regulation. However, several studies have shown distinct roles for NFAT proteins in the regulation of cell death. The TAD-C shows low sequence similarity among NFAT family members, but its contribution to specific NFAT1-induced phenotypes is poorly understood. Here, we described at least two regions of NFAT1 TAD-C that confer pro-apoptotic activity to NFAT1. These regions extend from amino acids 699 to 734 and 819 to 850 of NFAT1. We also showed that the NFAT1 TAD-C is unable to induce apoptosis by itself and requires a functional DBD. Furthermore, we showed that when fused to NFAT1 TAD-C, NFAT2, which is associated with cell transformation, induces apoptosis in fibroblasts. Together, these results suggest that the NFAT1 TAD-C includes NFAT death domains that confer to different NFAT members the ability to induce apoptosis.

## Introduction

The proteins belonging to the nuclear factor of activated T cells (NFAT) family of transcription factors were first reported to play a central role in transcription during immune responses. However, after the isolation and characterization of each protein of the NFAT family, it became clear that their expression was not restricted to T cells. At least one member of the NFAT family is expressed by almost every cell type that has been examined, including immune and non-immune cells [1]. The NFAT family consists of four calcium-responsive proteins named NFAT1 (NFATc2/NFATp), NFAT2 (NFATc1/NFATc), NFAT3 (NFATc4) and NFAT4 (NFATc3/NFATx). The regulation of NFAT by calcium influx is mediated by the NFAT homology region (NHR) [2]. This region is highly phosphorylated in resting cells, maintaining NFAT in an inactivated state in the cytoplasm [2]. Through sustained increases of intracellular calcium, NFAT is activated by calcineurin-mediated dephosphorylation and translocates to the nucleus. NFAT proteins also contain a highly conserved DNA-binding domain (DBD), which mediates binding to the DNA core sequence (A/T)GGAAA(A/N)(A/T/C)N [2]. In addition to the DNA-binding and regulatory domains, NFAT proteins include two transactivation domains (TAD) located at the N- and C-termini [2], [3]. These regions show relatively low sequence conservation among NFAT family members and may confer different regulatory ability of gene expression.

NFAT transcription factors are well-characterized for their role in the regulation of genes related to immune responses, such as interleukin-2 (IL-2), IL-4, IL-13, IL-21, IL-22, GM-CSF and interferon-γ [2], [4]. However, NFAT proteins have also been reported to regulate a wide range of genes involved in cell differentiation [5], cell cycle [6], [7], [8] and apoptosis [9], [10], [11]. Due to their high sequence conservation in the NHR and DBD, NFAT members play some overlapping roles in gene regulation [12], [13]. Nevertheless, non-redundant roles are evident in the phenotypes observed in individual NFAT knockout mice. Six-month old NFAT1 deficient mice show lymphocyte hyper-proliferation and an increased size of lymphoid organs [14], [15], [16]. In addition, mice lacking NFAT1 show retarded thymic involution and a reduction in the deletion of activated CD4^+^ T cells, indicating a possible defect in activation-induced cell death (AICD) [16]. In contrast, NFAT2 deficient mice die before day 14.5 of gestation due to a failure to develop normal cardiac valves and septa [17], [18]. However, in the RAG-2^−/−^ complementation system, NFAT2 deficient T cells show impaired proliferation and secretion of IL-4 [19]. The divergent roles of NFAT members have been further characterized elsewhere [20]. It has previously been shown that NFAT1 and NFAT2 have opposing effects in tumorigenesis [20]. The constitutively active form of NFAT1 (CA-NFAT1) induces cell cycle arrest and apoptosis in NIH3T3 fibroblasts and inhibits H-rasV12-induced transformation [20]. On the other hand, CA-NFAT2 induces cell transformation and tumor growth in allograft models [20], [21]. Interestingly, the long C-terminus TAD (TAD-C) of NFAT1 was shown to be essential for the divergent phenotypes induced by NFAT1 and NFAT2 [20]. In fact, the induction of apoptosis by NFAT1 in NIH3T3 fibroblasts is dependent on its TAD-C [20].

The NFAT1 TAD-C is composed of 248 amino acid residues and extends from amino acid residue 679 to 927. Here, we investigated the amino acid residues and putative domains of NFAT1 TAD-C that are required for NFAT1-induced apoptosis in NIH3T3 fibroblasts. We found at least two regions of the NFAT1 TAD-C that confer the pro-apoptotic activity of NFAT1. These regions comprise amino acids 699 to 734 and 819 to 850 from NFAT1. We also showed that NFAT1 TAD-C is unable to induce apoptosis by itself and requires a functional NFAT DBD. Furthermore, we showed that when fused to NFAT1 TAD-C, NFAT2, an NFAT member associated with cell transformation [20], [21], induces apoptosis in fibroblasts. Together, these results suggest that the NFAT1 TAD-C includes NFAT death domains that mediate the induction of apoptosis by different NFAT members.

## Materials and Methods

### Cell Culture

NIH3T3 cells (ATCC, Manassas, VA) were maintained in Dulbecco’s modified medium supplemented with 10% fetal bovine serum (FBS), L-glutamine, penicillin-streptomycin, essential and nonessential amino acids, sodium pyruvate, vitamins, HEPES and β-mercaptoethanol (All from Invitrogen, Carlsbad, CA) at 37°C and 5% CO_2_ in a humidified environment.

### Plasmid Construction

The retroviral expression vector pLIRES-EGFP was used to express CA-NFAT1 (Figure S1), CA-NFAT2 (Figure S1), CA-NFAT1-MutDBD, CA-NFAT2-TAD-C-NFAT1 chimera and each CA-NFAT1 construct described below. The plasmids pLIRES-EGFP, pLIRES-EGFP-CA-NFAT1, pLIRES-EGFP-CA-NFAT1Δ699-927 (pLIRES-EGFP-CA-NFAT1ΔC) and pLIRES-EGFP-CA-NFAT2/α have been previously described [20], [22]. To clone the CA-NFAT1 constructs CA-NFAT1Δ759-927, CA-NFAT1Δ819-927 and CA-NFAT1Δ889-927 with TAD-C deletions into the retroviral vector pLIRES-EGFP, we amplified portions of CA-NFAT1 by PCR (to amino acid residues 758, 818 or 888, respectively) using an anti-sense primer adapted with an EcoRV restriction site. The amplicon was cleaved by XhoI and EcoRV and inserted into pLIRES-EGFP-CA-NFAT1 cleaved by XhoI and NruI. These constructs were made using pLIRES-EGFP-CA-NFAT1 as a backbone to maintain the nuclear localization signal of the SV40 T antigen located at the C-terminus. The CA-NFAT1 constructs CA-NFAT1Δ699-758, CA-NFAT1Δ699-818, CA-NFAT1Δ699-850, CA-NFAT1Δ699-888 and CA-NFAT1Δ735-850 were constructed by inserting an HpaI restriction site within CA-NFAT1 at two points of interest corresponding to the amino acid positions specified in their names with the GeneTailor Site Directed Mutagenesis system (Invitrogen, Carlsbad, CA). Then, the mutated CA-NFAT1 was cleaved by HpaI and ligated, thereby deleting the amino acids residues between the HpaI sites. NFAT1 691-927 cDNA was amplified by PCR, using CA-NFAT1 as a template and a sense primer adapted with a new initiation codon. Later, this amplicon was inserted into the retroviral vector pLIRES-EGFP. The CA-NFAT2 TAD-C NFAT1 chimera was constructed in two steps. First, the NFAT1-TAD-C cDNA was amplified by PCR using pLIRES-EGFP-CA-NFAT1 as template and a sense primer adapted with an XhoI restriction site and cloned into the retroviral vector pLIRES-EGFP, creating pLIRES-EGFP-NFAT1-TAD-C. Second, the CA-NFAT2 cDNA was amplified by PCR until the end of its DBD using pLIRES-EGFP-CA-NFAT2/α as a template and an anti-sense primer adapted with an XhoI restriction site and cloned in-frame into pLIRES-EGFP-NFAT1-TAD-C, constructing pLIRES-EGFP-CA-NFAT2-TAD-C-NFAT1. The CA-NFAT1-MutDBD was constructed by mutating the amino acids residues of arginine, tyrosine and glutamate in positions 423, 426 and 429, respectively, to alanine, using the GeneTailor Site-Directed Mutagenesis system (Invitrogen). These amino acid residues from the highly conserved recognition loop present in the DBD have been shown to be essential for DNA contact [23]. All of the constructs were confirmed by restriction enzyme mapping and DNA sequencing. All of the primer sequences are available upon request.

### Production of Recombinant Retroviruses and Infection of NIH3T3 Cells

The BD EcoPack2 ecotropic packaging cell line (BD Biosciences, San Jose, CA) was transiently transfected with a retroviral vector by calcium phosphate precipitation for 24 h. The virus-containing cell supernatant was collected 48 h after transfection, supplemented with 8 µg/ml Polybrene (Fluka Chemie, Buchs, Switzerland) and immediately used for spin infection (twice for 45 min each time at 400×g at room temperature) of 2.5×10^4^ NIH3T3 cells. The infected cells were incubated at 37°C for an additional 24 h and trypsinized, and the efficiency of transduction was assessed by enhanced green fluorescent protein (EGFP) expression using flow cytometry analysis. For the CA-NFAT1 and NFAT1 691-927 co-expression experiments, cells were first infected with NFAT 691-927 expression vector, and 48 h after the infection, co-infected with CA-NFAT1 or empty vector. The efficiency of transduction was analyzed for NFAT1 691-927 expression by EGFP expression prior to the second round of infection and for CA-NFAT1 using intracellular staining with the NFAT1 polyclonal antibody anti-67.1 [24] and rhodamine-labeled anti-rabbit immunoglobulin G (KPL, Gaithersburg, MD) by flow cytometric analyses. To ensure reproducibility, each experiment was repeated using cells derived from independent viral infections.

### Cell Proliferation Assay

To assess proliferation, 8×10^3^ NIH3T3 wild-type cells infected with either the control pLIRES-EGFP or pLIRES-EGFP-NFAT construct virus were plated in triplicate in 96-well microtiter plates. Cell proliferation was analyzed at the indicated times by crystal violet. The crystal violet incorporation assay was performed by fixing the cells with ethanol for 10 min, followed by staining them with 0.05% crystal violet in 20% ethanol for 10 min and solubilization with methanol. The plate was read on a spectrophotometer at 595 nm (SpectraMax 190, Molecular Devices, Sunnyvale, CA).

### Cell Cycle and Sub-G0 Analysis

To assess the cell cycle and the sub-G0 DNA content, 2.4×10^4^ or 2.4×10^5^ NIH3T3 cells were plated in six-well microtiter plates, respectively. On the indicated day, the cells were trypsinized and washed once with phosphate-buffered saline (PBS). The cells were then stained with propidium iodide (75 µM) in the presence of NP-40. Analysis of the DNA content was performed by collecting 10,000 events for cell cycle analysis or 15,000 events for sub-G0 analysis using a FACScalibur flow cytometer and CellQuest software (BD Biosciences, San Jose, CA).

### Pyknotic Nuclei Formation Analysis

NIH3T3 cells infected with either pLIRES-EGFP or pLIRES-EGFP-CA-NFAT1 viruses were fixed with paraformaldehyde (4%) at room temperature for 15 minutes. Then, the cells were incubated in wash buffer (PBS 1x, 0.5% NP-40, 5% FBS) for one hour for membrane permeabilization. Later, the cells were incubated in DAPI solution (300 nM) for 1 min and visualized with an Olympus BX60 fluorescence microscope.

### Annexin-V Staining

To assess the level of exposed phosphatidylserine, 2.4×10^5^ NIH3T3 cells infected with either pLIRES-EGFP or pLIRES-EGFP-NFAT virus were plated in six-well microtiter plates, trypsinized 24 h later, washed with PBS, stained with APC-conjugated annexin-V (BD Biosciences, San Jose, CA), and analyzed by flow cytometry.

### Electrophoretic Mobility Shift Assay (EMSA)

The NFAT1 DBD recombinant protein was expressed as previously described [23]. The NFAT1 mutated DNA-binding domain (MutDBD) was expressed following the same protocol used for the wild type DBD. The DBD and MutDBD were then purified under native conditions with Ni-NTA spin columns according to the manufacturer’s instructions (Qiagen, West Sussex, United Kingdom). The proteins were eluted in 50 mM Tris-Cl pH 8.0, 100 mM NaCl and 300 mM imidazole. Oligonucleotide duplexes (5.0 µg of each oligo) were generated by denaturation for 10 min at 95°C in hybridization buffer (10 mM Tris pH 7.5, 50 mM NaCl) followed by overnight hybridization at room temperature. Oligonucleotides (50 ng) were labeled for 1 hour at 37°C with 10 U T4 polynucleotide kinase (New England Biolabs, Ipswich, MA) and 50 µCi [γ^32^P] dATP (GE Healthcare, Little Chalfont, United Kingdom). Later, probes were purified with MicroSpin™ G-25 columns (GE Healthcare). Increasing amounts of purified NFAT1 DBD or NFAT1 MutDBD protein (10 nm, 100 nM, 500 nM and 1 µM) were incubated with the indicated labeled oligonucleotides (20,000 counts/min) and 0.2 µg/reaction of poly(dI:dC) (Amersham Biosciences) in a total volume of 20 µL of binding buffer (10 mM HEPES pH 7.0, 125 mM NaCl, 10% glycerol, 0.25 mM DTT, 0.8 mg/mL BSA) for 20 min at room temperature. DNA-protein complexes were separated by electrophoresis under nondenaturing conditions on a 4% polyacrylamide gel in 1x TBE buffer. Later, the gel was dried onto Whatman filter paper and analyzed by autoradiography. The following oligonucleotide was used for the IL-2 promoter: 5′ GCCCAAAGAGGAAAATTTGTTTCATACAG 3′.

### Transactivation Assay

Jurkat cells (2×10^6^ cells/600 µl) were electroporated (950 µF, 250 V) in a 0.4 cm GenePulser Cuvette with GenePulser II (Bio-Rad Laboratories, Hercules, CA). The cells were co-transfected with three different plasmids in serum-free media, as follows: (1) the indicated retroviral vectors (10 µg), (2) the pGL4.30 reporter plasmid (2 µg) (Promega, Madison, WI), and (3) the pRL-TK *Renilla* expression plasmid for normalization (0.2 µg) (Promega). After 24 hours, cells were harvested and lysed for 15 min at room temperature with 50 µl of 1x cell culture lysis reagent (Promega). Crude extracts (20 µl) were added to 30 µl of luciferase assay substrate (Promega), and luciferase activity was promptly measured in a Veritas Microplate Luminometer (Promega). Luciferase activities were expressed as relative light units (RLU).

### Western Blot

Total protein from 4×10^5^ NIH3T3 cells was obtained from cells lysis in buffer containing 40 mM Tris pH 7.5, 60 mM sodium pyrophosphate, 10 mM EDTA, and 5% SDS, followed by incubation at 100°C for 15 min. Total cell lysates were resolved by SDS-PAGE, and the separated proteins were transferred onto a nitrocellulose membrane. The antibodies used were as follows: GAPDH monoclonal antibody 6C5 (Santa Cruz Biotechnology, Santa Cruz, CA), NFAT1 polyclonal antibody anti-67.1 [24] or anti-T2B1 [25] and NFAT2 monoclonal antibody 7A6 (Santa Cruz Biotechnology, Santa Cruz, CA). Immunodetection was performed with the ECL Western Blotting Detection Kit (GE Healthcare).

## Results

### CA-NFAT1 Expression in NIH3T3 Fibroblasts Induces Apoptosis

To study the role of the NFAT1 TAD-C in NFAT1-induced cell death, we used the previously described constitutively active form of NFAT1 isoform C (CA-NFAT1) [20], [26]. CA-NFAT1 has several mutations of serine residues to alanine in the NHR that prevent its phosphorylation and consequent inactivation (Figure S1). Expression analysis showed that the CA-NFAT1 is restricted to the nucleus and is able to transactivate an NFAT responsive promoter in a luciferase gene-reporter assay allowing the analysis of the phenotype induced by CA-NFAT1 in the absence of external stimuli (data not shown). Retroviral transduction was used to introduce CA-NFAT1 into NIH3T3 cells, and >80% transduction was regularly achieved (data not shown).

**Figure 1:**
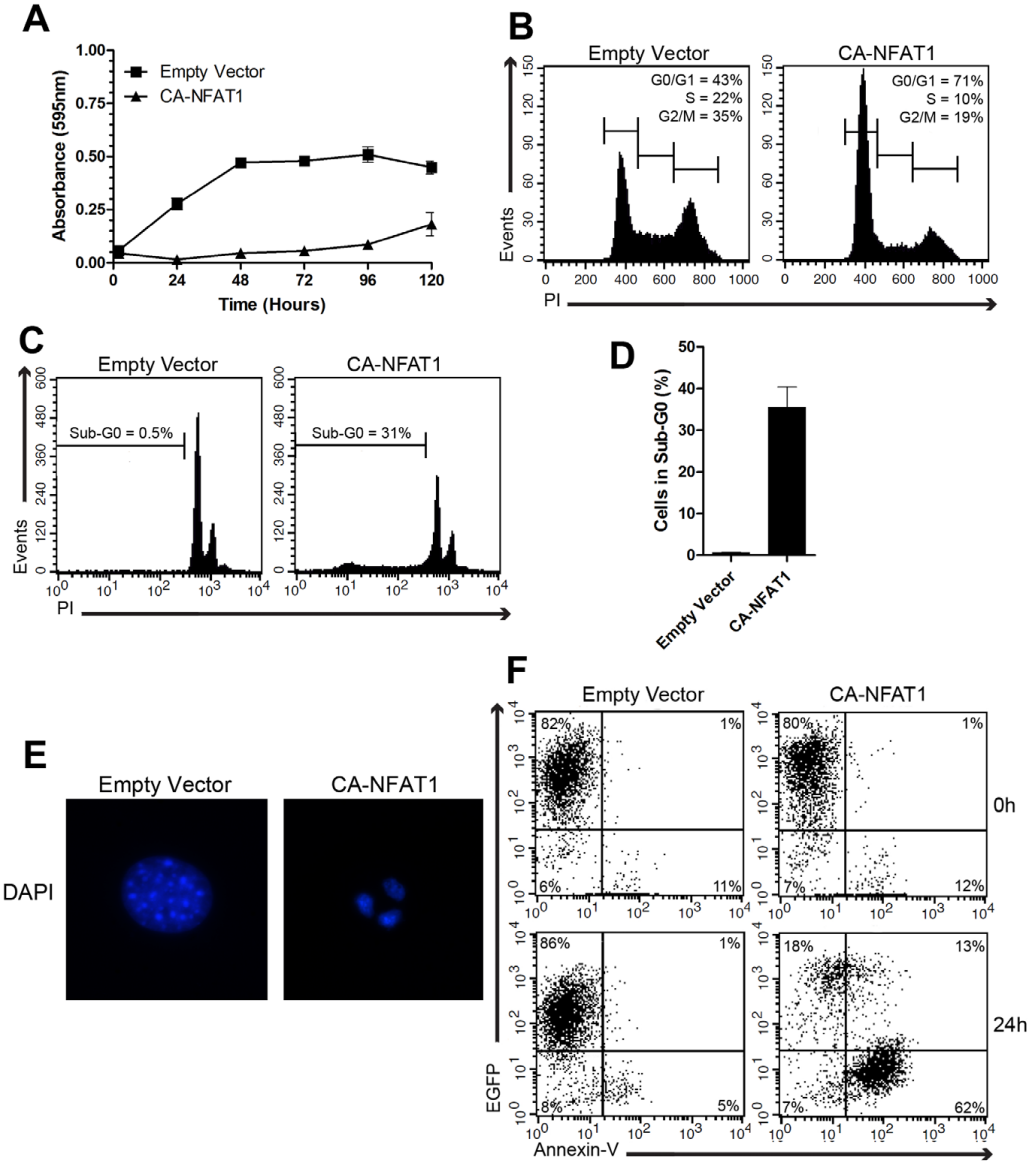
CA-NFAT1 expression induces cell cycle arrest and apoptosis in NIH3T3 fibroblasts. NIH3T3 cells were transduced with empty vector or retrovirus expressing CA-NFAT1 and subjected to proliferation, cell death and cell cycle assays. (A) Proliferation was assessed by incorporation of crystal violet. The cells were plated in triplicate and analyzed for proliferation for 120 hours. This graph is representative of three independent experiments. (B, C, D) NIH3T3 cells were stained with propidium iodide (PI) and analyzed by flow cytometry for cell cycle and death. (B) Cell cycle was analyzed by the incorporation of propidium iodide (PI). The cells were plated in triplicate and analyzed 24 hours after plating. The percentage of cells in each phase of the cell cycle is indicated in the graphs. (C) Analysis of cell death 48 hours after plating. The percentage of cells in sub-G0 is shown in the graph. (D) The graph shows the average percentage of cells in sub-G0 from three independent experiments. (E) Analysis of pyknotic nuclei by DAPI staining. Cells were stained 48 hours after plating and visualized by fluorescence microscopy. (F) Analysis of phosphatidylserine exposure 24 hours after plating. The cells were stained with APC-conjugated annexin-V and analyzed by flow cytometry. The graph showing annexin-V staining and EGFP fluorescence is representative of three independent experiments.

To evaluate the induction of apoptosis by NFAT1, NIH3T3 cells were infected with empty vector (control) or a CA-NFAT1-expressing vector. First, proliferation was assessed by crystal violet incorporation, which correlates with total cell number. While NIH3T3 cells transduced with empty vector proliferated until confluence, CA-NFAT1-expressing cells had low proliferation levels and showed a reduction in total cell number (Figure 1A). Next, to assess whether the low proliferation levels observed in CA-NFAT1-expressing NIH3T3 cells reflects a reduction in the number of cells entering the cell cycle, we evaluated the cell cycle profile 24 hours after plating by propidium iodide staining. Cell cycle analysis revealed that CA-NFAT1 expression arrested cells at G0/G1 phase of the cell cycle (Figure 1B).

In addition to a low proliferation rate, we also observed a reduction in the total number of CA-NFAT1-expressing cells. Thus, we next assessed the presence of apoptotic features in NIH3T3 cells, as NFAT1 has been reported to induce the expression of apoptotic genes [9], [10], [11]. The sub-G0 DNA content of NIH3T3 cells infected with the empty vector or CA-NFAT1-expressing vector was evaluated by propidium iodide staining to examine DNA fragmentation. As shown in Figures 1C and 1D, control cells showed a low percentage of cells with sub-G0 DNA content. On the other hand, 48 hours after plating, approximately 30% of the CA-NFAT1-expressing cells had sub-G0 DNA content (Figure 1C and 1D), suggesting that CA-NFAT1 expression induces apoptosis of NIH3T3 fibroblasts. To better characterize the phenotype induced by CA-NFAT1, we assessed pyknotic nuclei formation, another indication of apoptosis. In agreement with the sub-G0 DNA content analysis, we observed pyknotic nuclei formation in CA-NFAT1-expressing NIH3T3 cells, while the control cells did not contain pyknotic nuclei as determined by fluorescence microscopy (Figure 1E). Furthermore, the expression of CA-NFAT1 in NIH3T3 cells resulted in high phosphatidylserine exposure, another well-known feature of apoptosis (Figure 1F). Approximately 75% of the CA-NFAT1-expressing cells were positive for annexin-V 24 hours after plating (Figure 1F). In contrast, a low percentage of cells infected with empty retrovirus was positive for annexin-V (Figure 1F). Together, these data suggest that CA-NFAT1 induces cell cycle arrest and apoptosis in NIH3T3 fibroblasts.

### The Deletion of Amino Acid Residues 699 to 850 of the NFAT1 TAD-C Abolishes CA-NFAT1-induced Apoptosis

The NFAT transactivation domains are not well conserved among NFAT family members [2], and the NFAT1 C-terminus transactivation (NFAT1 TAD-C) domain has been shown to be responsible for some of the differences of NFAT-mediated gene regulation [11], [27], [28]. Indeed, we have previously shown that the ability of CA-NFAT1 to induce apoptosis is dependent on its TAD-C [20]. However, it is not known which amino acid residues of the TAD-C are required for CA-NFAT1-induced apoptosis. To better understand the role of the TAD-C in NFAT1-induced apoptosis, we mapped the TAD-C amino acids residues required by NFAT1 to induce apoptosis. To this end, we constructed several CA-NFAT1 truncated proteins that lack different regions of the NFAT1 TAD-C. The CA-NFAT1 truncated proteins were named according to the amino acid residues deleted. All of the CA-NFAT1 truncated protein showed similar expression levels and had the expected molecular weight (Figure S2).

**Figure 2:**
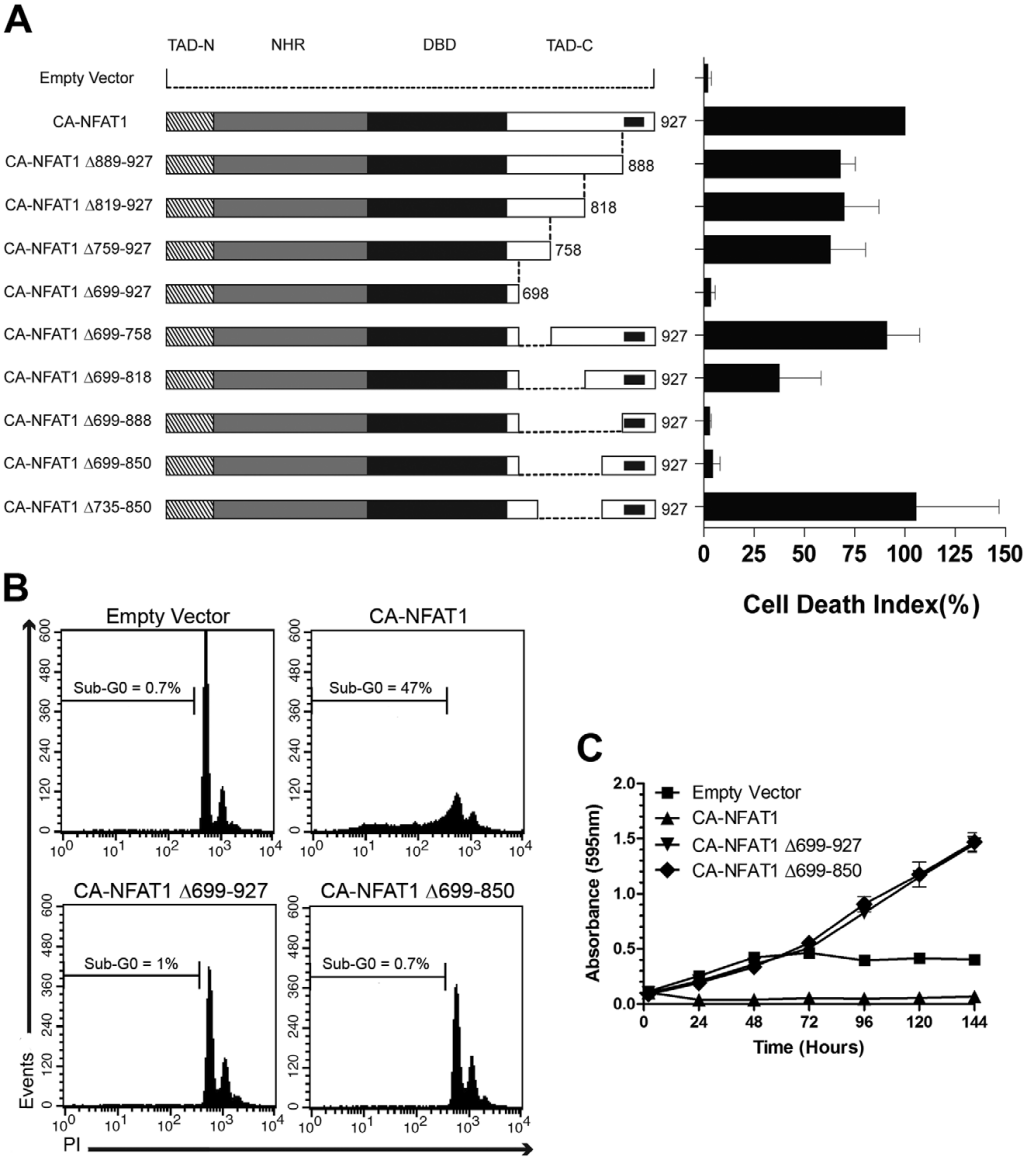
The deletion of amino acids 699 to 850 of TAD-C prevents the induction of apoptosis by CA-NFAT1. NIH3T3 cells were transduced with empty vector or retrovirus expressing CA-NFAT1 or the indicated CA-NFAT1 truncated proteins. (A, B) NIH3T3 cells were stained with propidium iodide (PI) and analyzed by flow cytometry for cell death. (A) Analysis of cell death 48 hours after plating. The graph shows the average levels of cell death observed in three independent experiments. This graph was normalized by setting the percentage of cells with sub-G0 DNA content induced by CA-NFAT1 to 100%. The cell death index shown is the ratio of the percentage of cells in sub-G0 induced by empty vector or the indicated CA-NFAT1 construct and the percentage of cells in sub-G0 induced by full-length CA-NFAT1. A schematic of full-length CA-NFAT1 and the truncated CA-NFAT1 proteins is shown. The crosshatched bar represents TAD-N (N-terminal transactivation domain), the grey bar represents NHR (NFAT-homology region), the black bar represents the DBD (DNA-binding domain), and the white bar represents the TAD-C (C-terminal transactivation domain). (B) Representative graph of sub-G0 DNA content of NIH3T3 cells transduced with empty vector or retrovirus expressing CA-NFAT1, CA-NFAT1 Δ699-927 or CA-NFAT1 Δ699-850. The percentage of cells in sub-G0 is shown in the graph. (C) Proliferation was assessed by incorporation of crystal violet. The cells were plated in triplicate and analyzed for 144 hours. This graph is representative of three independent experiments.

**Figure 3:**
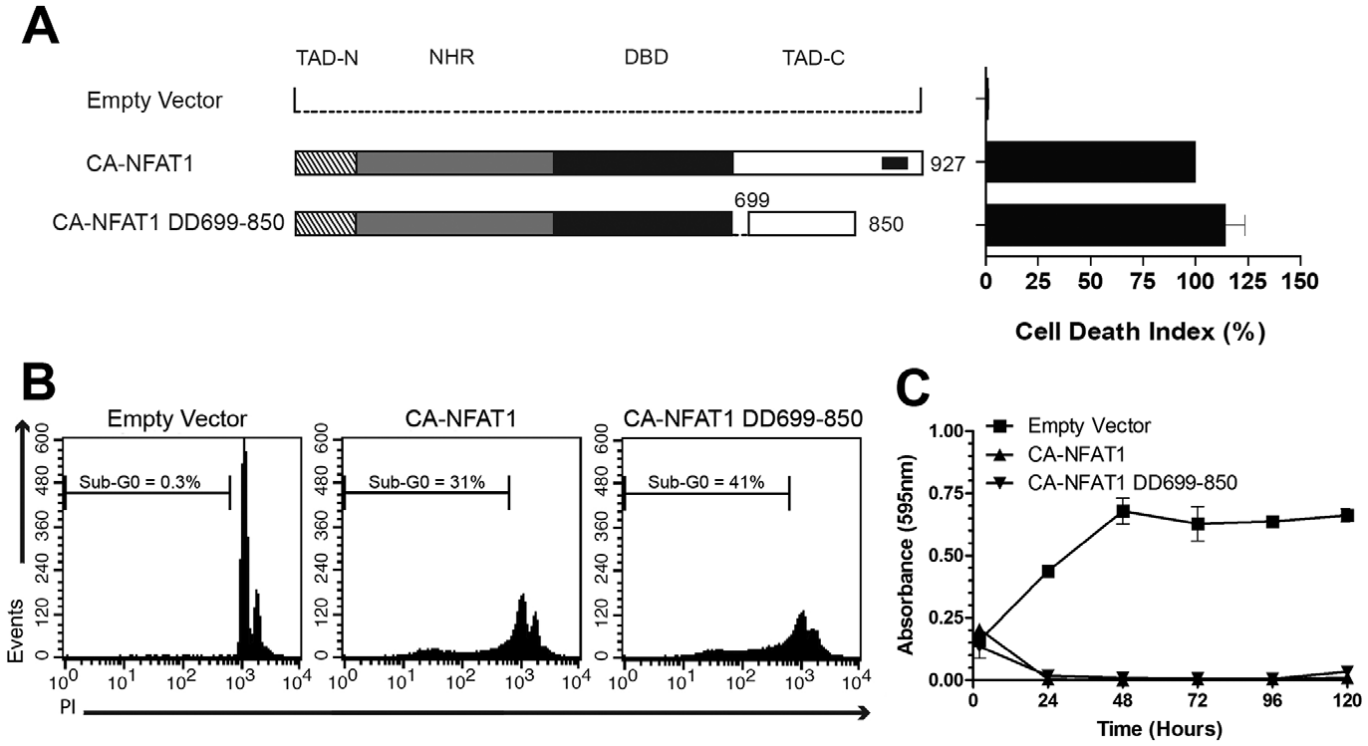
The amino acids 699 to 850 of TAD-C are sufficient to provide to CA-NFAT1 the ability to induce apoptosis. NIH3T3 cells were transduced with empty vector or retrovirus expressing CA-NFAT1 or CA-NFAT1 DD699-850. (A, B) NIH3T3 cells were stained with propidium iodide (PI) and analyzed by flow cytometry for cell death. (A) Analysis of cell death 48 hours after plating. The graph shows the average levels of cell death observed in three independent experiments. This graph was normalized by setting the percentage of cells with sub-G0 DNA content induced by CA-NFAT1 to 100%. The cell death index shown is the ratio of the percentage of cells in sub-G0 induced by empty vector or the indicated CA-NFAT1 construct and the percentage of cells in sub-G0 induced by full-length CA-NFAT1. A schematic of full-length CA-NFAT1 and CA-NFAT1 DD699-850 proteins is shown. (B) Representative graph of sub-G0 DNA content of NIH3T3 cells transduced with empty vector or retrovirus expressing CA-NFAT1 or CA-NFAT1 DD699-850. The percentage of cells in sub-G0 is shown in the graph. (C) Proliferation was assessed by incorporation of crystal violet. The cells were plated in triplicate and analyzed for 120 hours. This graph is representative of three independent experiments.

To evaluate the ability of these CA-NFAT1 truncated proteins to induce apoptosis, we performed sub-G0 DNA content analysis of NIH3T3 cells expressing each construct. First, we observed that CA-NFAT1Δ699-927, which lacks the TAD-C, completely lost the ability to induce apoptosis, in contrast to full length CA-NFAT1, indicating that the NFAT1 TAD-C is essential for induction of apoptosis by CA-NFAT1 in our model (Figure 2A and 2B). On the other hand, CA-NFAT1Δ759-927 induced apoptosis, similar to CA-NFAT1, indicating that the amino acid residues required for CA-NFAT1-mediated apoptosis are between amino acids 699 to 758 (Figure 2A). However, the truncated protein CA-NFAT1Δ699-758, which lacks amino acids 699 to 758, was still able to induce apoptosis, suggesting the existence of multiple regions of the NFAT1 TAD-C that play a role in NFAT1-mediated apoptosis (Figure 2A). This hypothesis was supported by the phenotypes induced by CA-NFAT1Δ699-850 and CA-NFAT1Δ699-818. CA-NFAT1Δ699-850 failed to induce apoptosis in NIH3T3 cells, similar to CA-NFAT1Δ699-927 (Figure 2A and 2B). On the other hand, CA-NFAT1Δ699-818 induced a moderate degree of cell death when compared to the full-length CA-NFAT1, indicating that amino acids 819 to 850 also play a role in NFAT1-induced apoptosis (Figure 2A). Next, we added 35 amino acid residues from positions 699 to 734 to the CA-NFAT1Δ699-850 construct, creating CA-NFAT1Δ735-850. CA-NFAT1Δ735-850 induced high levels of apoptosis, similar to the full-length CA-NFAT1 (Figure 2A). Together, these results showed that the removal of amino acids 699 to 850 prevents the induction of apoptosis by CA-NFAT1 and suggest that there are at least two domains within TAD-C, between amino acids 699 to 734 and 819 to 850, which mediate CA-NFAT1-induced apoptosis. To further investigate the role of these two domains located between amino acids 699 and 850, we constructed a CA-NFAT1 truncated protein containing only amino acids 699 to 850 from TAD-C (CA-NFAT1 DD699-850) and assessed its ability to induce apoptosis by sub-G0 DNA content analysis. When expressed in NIH3T3 fibroblasts, CA-NFAT1 DD699-850 induced apoptosis similarly to CA-NFAT1 (Figure 3A and 3B). CA-NFAT1 DD699-850 expression also reduced proliferation in a CA-NFAT1-like manner (Figure 3C). Therefore, these results suggest that this region of TAD-C plays a critical role in NFAT1-induced apoptosis.

We also assessed whether there are different roles in apoptosis for NFAT1 splice variants that are divergent in their TAD-C. NFAT1 isoforms B and C are the predominant isoforms in T cells [25]. NFAT1 isoform B diverges in its TAD-C from NFAT1 isoform C at amino acid residue 907. Despite this divergence, sub-G0 DNA content analysis indicated that the constitutively active form of NFAT1 isoform B induced apoptosis in NIH3T3 cells in a manner similar to CA-NFAT1 isoform C (data not shown). This result suggests that both NFAT1 isoforms B and C induce apoptosis and corroborates our conclusion that the NFAT1 TAD-C region required to induce apoptosis is located between amino acids 699 to 850. Moreover, we also evaluated the role of non-constitutively active form of NFAT1 protein on trigger apoptosis. As shown in Figure S3, wild-type NFAT1 was not able to induce apoptosis when expressed in NIH3T3 fibroblasts upon stimulation with phorbol-12-myristate-13-acetate (PMA) and ionomycin. However, we cannot rule out the role of NFAT1 protein in apoptosis induction, since the PMA plus ionomycin stimulation may trigger survival pathways that could prevent cell death [29]. Furthermore, this result may indicate that sustained activity of NFAT1 is required for induction of cell death.

In addition to the suppression of apoptosis, deletion of the NFAT1 TAD-C has been shown to induce hyper-proliferation in fibroblasts [20]. To assess whether the removal of amino acids 699 to 850 of CA-NFAT1 induces hyper-proliferation, NIH3T3 cells infected with empty vector, CA-NFAT1, CA-NFAT1Δ699-850 or CA-NFAT1Δ699-927 were analyzed for proliferation. This assay revealed that CA-NFAT1Δ699-927, which lacks the NFAT1 TAD-C, and CA-NFAT1Δ699-850, which fails to induce apoptosis, were able to induce hyper-proliferation of NIH3T3 cells (Figure 2B and 2C). The CA-NFAT1Δ699-927- and CA-NFAT1Δ699-850-expressing cells proliferated beyond confluence, while the CA-NFAT1-expressing cells did not (Figure 2C). Taken together, these results further support the role of amino acid residues 699 to 850 in NFAT1-induced apoptosis and suggest that there is a duality of NFAT1 that regulates both proliferation and cell death.

### Overexpression of the NFAT1 C-terminus Peptide is Unable to Prevent CA-NFAT1-mediated Apoptosis

**Figure 4:**
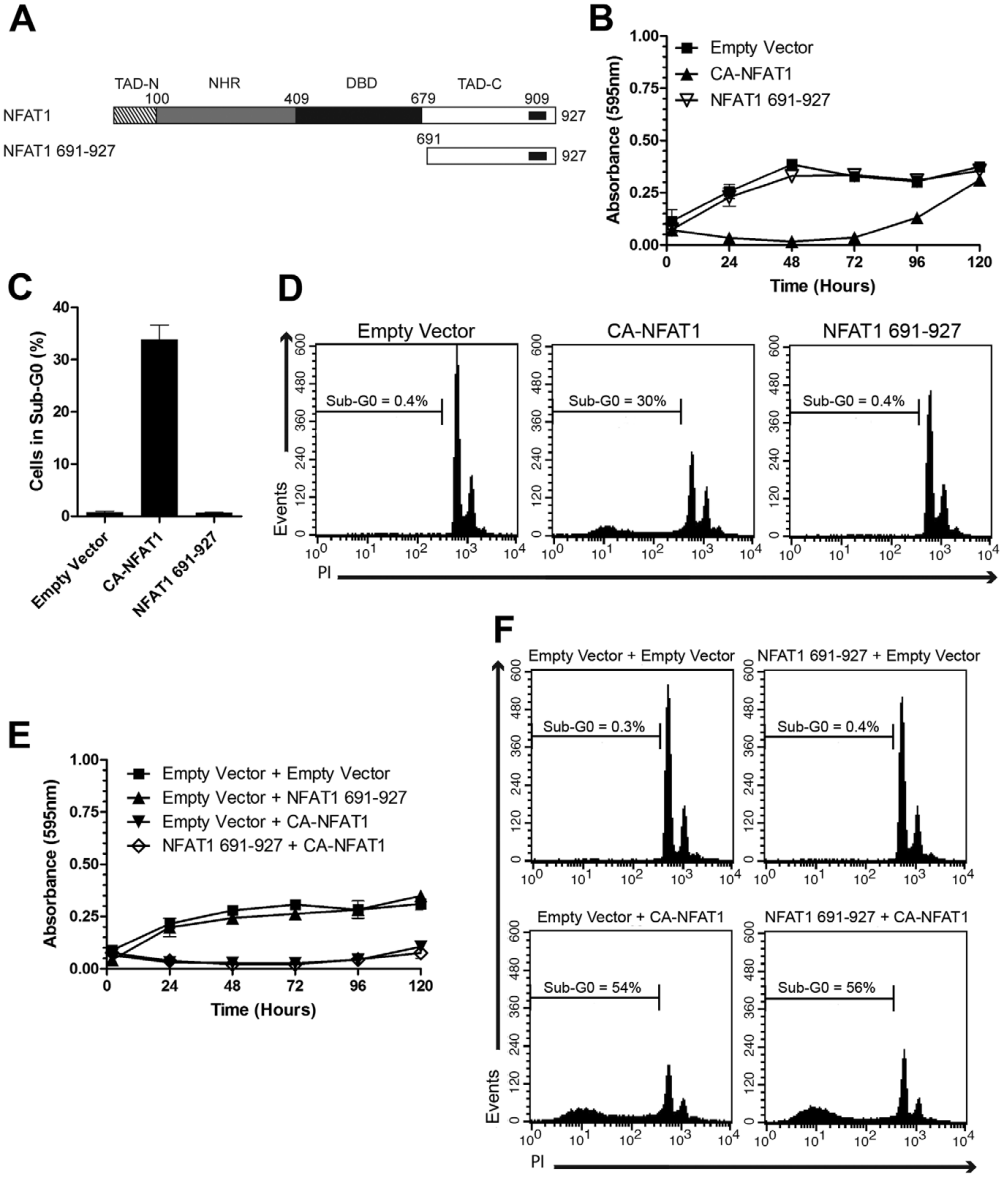
The overexpression of NFAT1 691-927 does not prevent the induction of apoptosis by CA-NFAT1. (A) Schematic representation of the primary structure of NFAT1 and NFAT1 691-927. See Figure 2A for detailed information. (B, C, D) NIH3T3 cells were transduced with empty vector or retrovirus expressing CA-NFAT1 or NFAT1 691-927. (B) Proliferation was assessed by incorporation of crystal violet. NIH3T3 cells were plated in triplicate and analyzed for 120 hours. This graph is representative of three independent experiments. (C, D) NIH3T3 cells were stained with propidium iodide (PI) and analyzed for cell death by flow cytometry. (C) Analysis of cell death 48 hours after plating. The graph shows the average percentage of cells in sub-G0 determined in three independent experiments. (D) Representative graph of cell death analysis shown in (C). The percentage of cells in sub-G0 is shown in the graph. (E, F) NIH3T3 cells transduced with empty vector or retrovirus expressing NFAT1 691-927 were re-infected with empty vector or retrovirus expressing CA-NFAT1 and subjected to proliferation and cell death assays. (E) Proliferation was assessed by incorporation of crystal violet. The cells were plated in triplicate and analyzed for 120 hours. This graph is representative of three independent experiments. (F) Cell death analysis 48 hours after plating. NIH3T3 cells were stained with propidium iodide (PI) and analyzed by flow cytometry for cell death. The percentage of cells with sub-G0 DNA content is shown in the graph.

The apoptosis induced by CA-NFAT1 is dependent on amino acid residues 699 to 850 of the NFAT1 TAD-C (Figure 2), however, it is unclear whether the NFAT1 TAD-C alone can induce apoptosis. To assess this hypothesis, we constructed a retroviral vector expressing the NFAT1 C-terminus peptide, NFAT1 691-927. This protein was fused to the nuclear localization signal of the SV40 T antigen for constitutive localization in the nucleus (data not shown). A schematic alignment of NFAT1 and NFAT1 691-927 is shown in Figure 4A. Next, NIH3T3 cells were infected with empty vector or CA-NFAT1- or NFAT1 691-927-expressing vectors, and the cells were subjected to proliferation assays. We observed that the NFAT1 691-927-expressing cells and control cells proliferated until confluence, in contrast to the CA-NFAT1-expressing cells, indicating that NFAT1 691-927 is unable to induce apoptosis (Figure 4B). These results were later confirmed by sub-G0 DNA content analysis. The NFAT1 691-927-expressing cells had a low percentage of cells with sub-G0 DNA content, which was similar to control cells (Figure 4C and 4D). However, approximately 30% of the CA-NFAT1-expressing cells had sub-G0 DNA content 48 hours after plating (Figure 4C and 4D). Together, these data demonstrate that NFAT1 691-927 does not trigger apoptosis by itself, indicating that other CA-NFAT1 domains are required.

NFAT transcription factors interact with several transcription partners that are important integrators of the calcineurin/NFAT pathway with other signaling pathways [1]. The induction of apoptosis by CA-NFAT1 could be dependent on interaction between the NFAT1 TAD-C and an NFAT partner. Therefore, we hypothesized that NFAT1 691-927 may act as a dominant negative of CA-NFAT1 by competing for the binding to an NFAT partner. To test this hypothesis, NIH3T3 cells were infected with empty vector or the NFAT1 691-927 vector, and co-infected with empty vector or the CA-NFAT1-expressing vector 48 hours later. As shown in Figures 4E and 4F, the NIH3T3 cells that were co-infected with empty retrovirus and CA-NFAT1-expressing cells revealed low levels of proliferation and high levels of DNA fragmentation. Similarly, the NIH3T3 cells that were co-infected with CA-NFAT1 and NFAT1 691-927 vectors displayed low proliferation (Figure 4E) and high percentages of cells with sub-G0 DNA content (Figure 4F), indicating that NFAT1 691-927 does not act as a dominant negative of CA-NFAT1.

### The Induction of Apoptosis by CA-NFAT1 is Dependent on DNA Binding

**Figure 5:**
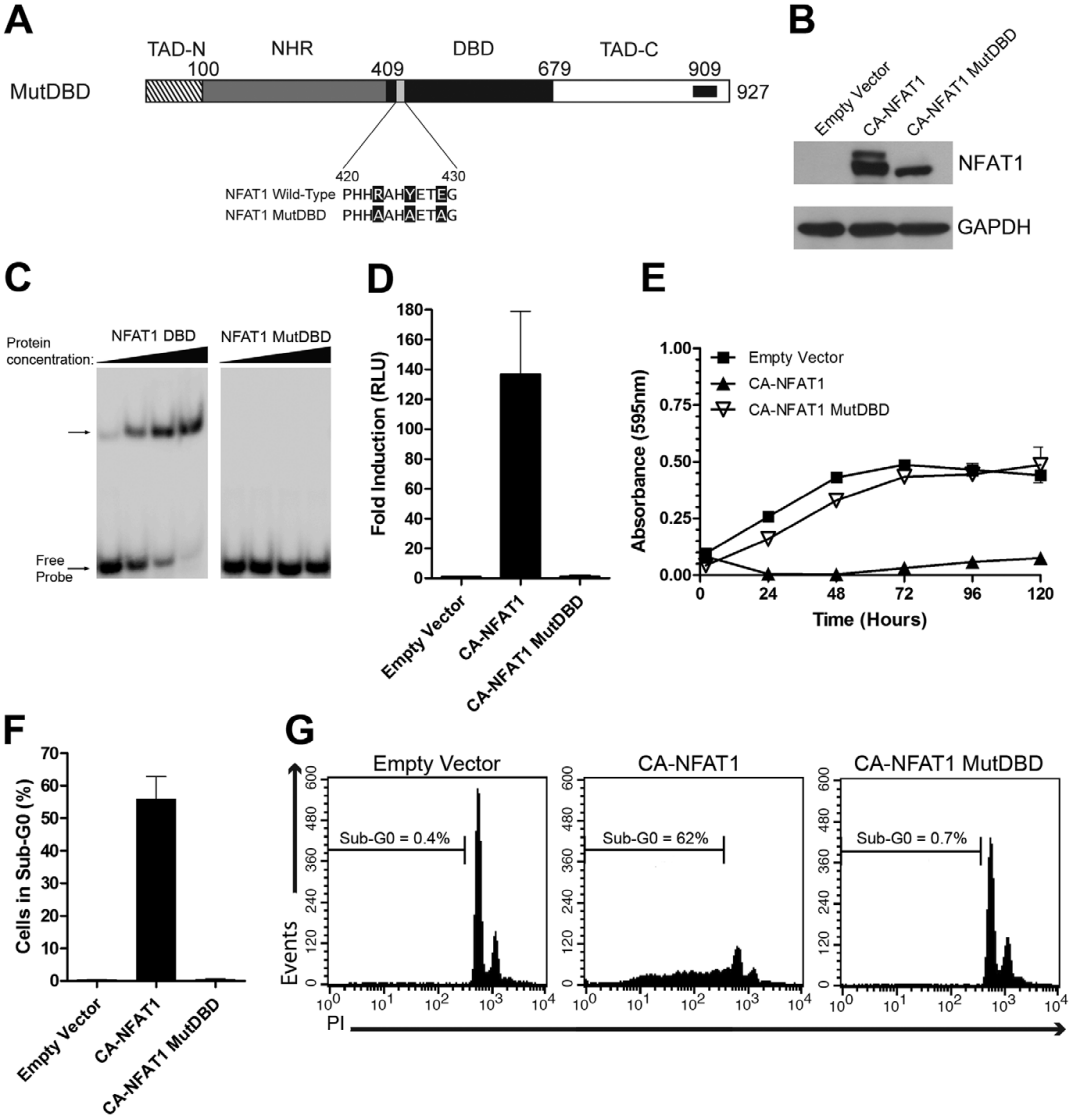
Apoptosis induced by CA-NFAT1 in NIH3T3 fibroblasts is dependent on DNA binding. (A) Schematic representation of the primary structure of CA-NFAT1 MutDBD. The mutated residues are indicated in the Figure. (B, E, F, G) NIH3T3 cells were transduced with empty vector or retrovirus expressing CA-NFAT1 or CA-NFAT1 MutDBD. (B) The total lysate of transduced NIH3T3 cells was obtained for analysis of NFAT1 and GAPDH expression levels by Western Blot. The molecular weights are indicated in kilodaltons (kDa). (C) The ability of the NFAT1 DBD and NFAT1 MutDBD to bind to DNA was tested by EMSA. The NFAT1 DBD and NFAT1 MutDBD peptides were incubated with oligonucleotides corresponding to the NFAT responsive element in the IL-2 promoter. (D) Jurkat cells were transfected with expression vector (empty vector or vector containing the CA-NFAT1 or CA-NFAT1 MutDBD cDNAs), luciferase reporter vector pGL4.30 and the Renilla luciferase expression vector pRL-TK. After 24 hours, the luciferase activity was measured by the release of luminescence resulting from the oxidation of its substrate (luciferin), normalized with the Renilla vector and expressed as relative light units (RLU). (E) Proliferation was assessed by incorporation of crystal violet. The cells were plated in triplicate and analyzed for 120 hours. This graph is representative of three independent experiments. (F, G) NIH3T3 cells were stained with propidium iodide (PI) and analyzed by flow cytometry for cell death. (F) Analysis of cell death 48 hours after plating. The graph represents the average percentage of cells in sub-G0 in three independent experiments. (G) Representative graph of the cell death analysis shown in (F). The percentage of cells in sub-G0 is shown in the graph.

The main function of the NFAT family members is to regulate the expression of genes. It has been postulated that NFAT proteins depend entirely on their ability to bind to DNA to regulate gene expression. Therefore, to test whether CA-NFAT1-induced apoptosis is dependent on NFAT1-mediated transcriptional activation, we constructed a CA-NFAT1 mutant that is unable to bind to DNA, CA-NFAT1 MutDBD. A schematic representation of CA-NFAT1 MutDBD is shown in Figure 5A. CA-NFAT1 MutDBD expression in NIH3T3 cells was confirmed by Western Blot (Figure 5B). Then, to confirm that the NFAT1 MutDBD cannot bind DNA, we performed an electrophoretic mobility shift assay. As shown in Figure 5C, wild-type NFAT1 DBD was able to bind to DNA in a dose-dependent manner, while NFAT1 MutDBD was unable to bind to DNA at any of the concentrations tested. Further, luciferase gene reporter assays were performed to confirm that the CA-NFAT1 MutDBD cannot activate gene expression. While CA-NFAT1 induced transactivation of an NFAT-responsive luciferase promoter that was 130-fold higher than that of the control, the empty vector and CA-NFAT1 MutDBD induced similarly low levels of transactivation, indicating that CA-NFAT1 MutDBD is unable to bind DNA and transactivate NFAT-responsive promoters (Figure 5D).

To assess whether CA-NFAT1 MutDBD induces cell death, NIH3T3 cells were infected with empty vector or CA-NFAT1 or CA-NFAT1 MutDBD vectors, and cell proliferation was assessed. As shown in Figure 5E, the CA-NFAT1-expressing cells showed a low rate of proliferation and a reduction in total cell number. However, the CA-NFAT1 MutDBD-expressing cells proliferated until confluence, similar to control cells (Figure 5E), demonstrating that CA-NFAT1 MutDBD is unable to induce cell death in NIH3T3 cells. To confirm this finding, the NIH3T3 cells infected with empty vector or CA-NFAT1 or CA-NFAT1 MutDBD vectors were assessed for sub-G0 DNA content by propidium iodide staining. The CA-NFAT1-expressing cells had a high percentage of cells with sub-G0 DNA content, while the control cells and CA-NFAT1 MutDBD-expressing cells showed low levels of sub-G0 DNA content, indicating that CA-NFAT1 MutDBD does not induce apoptosis in NIH3T3 cells (Figure 5F and 5G). Taken together, these results demonstrate that CA-NFAT1-mediated apoptosis in NIH3T3 cells requires DNA binding of NFAT1.

### Fusion of the NFAT1 TAD-C to CA-NFAT2 Confers the Ability to Induce Apoptosis

The NFAT transactivation domains have low conservation among NFAT family members and may confer different gene expression regulatory abilities. It has previously been shown that NFAT1 and NFAT2 have opposing effects on cell proliferation and death [20]. CA-NFAT1 induces cell cycle arrest and apoptosis in NIH3T3 fibroblasts [20], while CA-NFAT2 induces cell proliferation and protects cells from dying [20], [21]. The NFAT2 protein possesses a short TAD-C, and the long C-terminus of NFAT1 has been shown to be essential for the divergent phenotypes induced by NFAT1 and NFAT2 [20]. Thus, we assessed whether the NFAT1 TAD-C could induce apoptosis when fused to CA-NFAT2 and reverse the phenotype induced by CA-NFAT2 in NIH3T3 cells.

**Figure 6:**
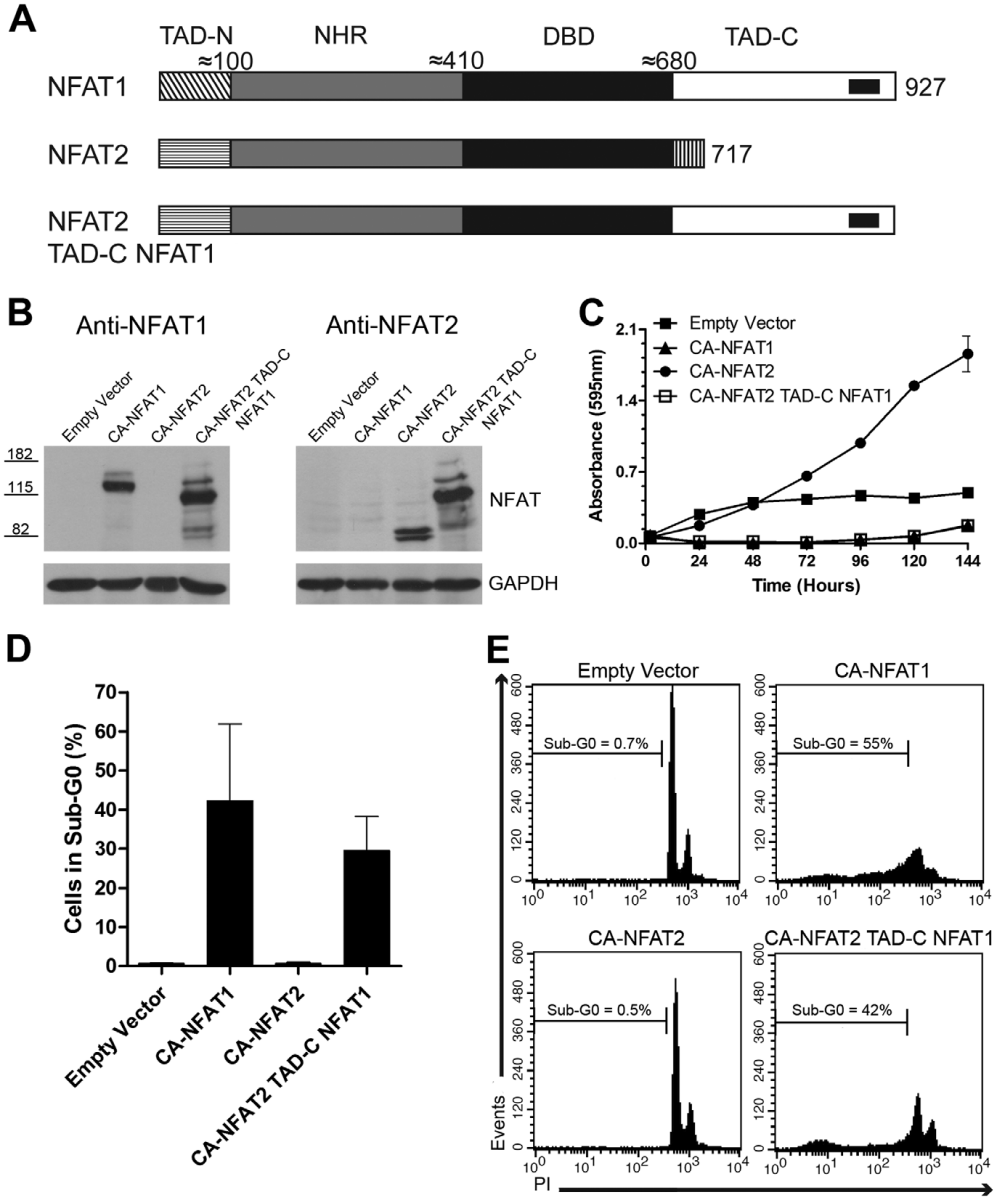
The fusion of NFAT1 TAD-C to CA-NFAT2 reverses the phenotype induced by CA-NFAT2. (A) Schematic representation of the primary structures of CA-NFAT1, CA-NFAT2 and CA-NFAT2 TAD-C NFAT1. See Figure 2A legend for details. (B, C, D, E) NIH3T3 cells were transduced with empty vector or retrovirus expressing CA-NFAT1, CA-NFAT2 or CA-NFAT2 TAD-C NFAT1. (B) The total lysate of transduced NIH3T3 cells was obtained for analysis of NFAT expression levels and molecular weight by Western Blot using anti-NFAT1 and anti-NFAT2 antibodies. Levels of the housekeeping protein GAPDH were also analyzed as a loading control. The molecular weights are indicated in kilodaltons (kDa). (C) Proliferation was assessed by incorporation of crystal violet. The cells were plated in triplicate and analyzed for 144 hours. This graph is representative of three independent experiments. (D, E) NIH3T3 cells were stained with propidium iodide (PI) and analyzed for cell death by flow cytometry. (D) Analysis of cell death 48 hours after plating. The graph shows the average percentage of cells in sub-G0 observed in three independent experiments. (E) Representative graph of cell death analysis shown in (D). The percentage of cells in sub-G0 is shown in the graph.

To this end, we fused the NFAT1 TAD-C to CA-NFAT2, creating the chimeric protein expression vector named CA-NFAT2-TAD-C-NFAT1. A schematic alignment of NFAT1, NFAT2 and the chimeric protein is shown in Figure 6A. Then, we performed Western Blot to assess whether the chimeric CA-NFAT2 protein includes the NFAT1 TAD-C. As shown in Figure 6B, the anti-NFAT1 antibody that recognizes the NFAT1 TAD-C recognized both CA-NFAT1 and CA-NFAT2-TAD-C-NFAT1 (left panel), and the anti-NFAT2 antibody detected the expression of CA-NFAT2 and CA-NFAT2-TAD-C-NFAT1 (right panel), indicating that the CA-NFAT2-TAD-C-NFAT1 chimeric protein is expressed in NIH3T3 cells.

Then, to assess whether CA-NFAT2-TAD-C-NFAT1 can induce cell death, NIH3T3 cells were infected with empty vector or CA-NFAT1-, CA-NFAT2- or CA-NFAT2-TAD-C-NFAT1-expressing vectors, and the cells were analyzed by proliferation assay and for sub-G0 DNA content. As shown in Figure 6C, the CA-NFAT2-expressing cells proliferated beyond confluence, in contrast to the CA-NFAT1-expressing cells. Interestingly, the CA-NFAT2-TAD-C-NFAT1-expressing cells had low rates of proliferation and a reduction in total cell number, similar to the CA-NFAT1-expressing cells (Figure 6C). These results demonstrated that CA-NFAT2-TAD-C-NFAT1 is able to induce a phenotype similar to that induced by CA-NFAT1 in NIH3T3 cells. We next determined whether this chimeric protein is also able to trigger apoptosis. Sub-G0 DNA content analysis revealed that CA-NFAT2-TAD-C-NFAT1 induced apoptosis similar to CA-NFAT1 (Figure 6D and 6E). In contrast, a low number of CA-NFAT2-expressing cells had sub-G0 DNA content (Figure 6D and 6E). Taken together, these results suggest that the NFAT1 TAD-C plus a functional NFAT DNA-binding domain confers the ability to induce apoptosis to an NFAT family member.

## Discussion

In this study, we showed that CA-NFAT1 expression induces apoptosis in NIH3T3 fibroblasts, as evidenced by cell features such as DNA fragmentation, pyknotic nuclei formation and phosphatidylserine exposure (Figure 1). Furthermore, our results identified a specific region of the NFAT1 TAD-C, amino acids 699 to 850, that is essential for the apoptosis induced by CA-NFAT1 (Figure 2 and 3). Although this region is still large, comprising 152 amino acids, our data suggest that the NFAT1 TAD-C includes at least two death domains between amino acids 699 to 734 and 819 to 850 that mediate the pro-apoptotic function of NFAT1 independently of other regions of TAD-C. Indeed, the truncated protein CA-NFAT1Δ699-850 was unable to induce apoptosis, while CA-NFAT1Δ735-850 and CA-NFAT1Δ699-818 induced apoptosis in NIH3T3 cells (Figure 2). Corroborating these results, CA-NFAT1 DD699-850, a CA-NFAT1 construct that contains only the TAD-C amino acids 699 to 850, induced apoptosis similarly to CA-NFAT1 (Figure 3). In addition, we also observed that CA-NFAT1Δ699-927 and CA-NFAT1Δ699-850 drove the NIH3T3 cells to proliferate beyond confluence (Figure 2C). This result suggests that NFAT1 can induce proliferation in the absence of the amino acid residues that are required for the induction of apoptosis. Interestingly, several studies have demonstrated that NFAT2 isoform α, an NFAT member that possess a short TAD-C, is involved in cell transformation [20], [21]. The constitutively active form of NFAT2 isoform α has been reported to induce colony formation, deregulation of contact inhibition and tumor growth in a mouse allotransplant tumor model [20], [21]. Taken together, these studies suggest that there is a dichotomy between NFAT members that is mediated by their TAD-C. To test this hypothesis, we analyzed a chimeric NFAT2 protein (CA-NFAT2-TAD-C-NFAT1). Interestingly, CA-NFAT2-TAD-C-NFAT1 reduced cell proliferation and induced apoptosis in NIH3T3 cells in a manner similar to CA-NFAT1 (Figure 6). Therefore, this result confirms that the NFAT1 TAD-C is a central mediator of the NFAT1 pro-apoptotic function.

To regulate gene expression, NFAT proteins interact with several proteins, such as transcriptional activators, co-activators and repressors [1]. Some of these interactions have been shown to be mediated by the TADs. Activator protein-1 (AP-1), the most well-characterized NFAT transcription partner, was first reported to interact with NFAT through its DBD [30]. However, functional interaction between AP-1 and NFAT1 has been shown to occur at the NFAT1 TAD-C [31]. NFAT1 also interacts with the IRF2BP2 transcriptional repressor through the TAD-C [28]. Moreover, the NFAT1 N-terminal TAD (TAD-N) interacts with other NFAT partners, such as p300, a well-known transcriptional co-activator [32]. Despite the low sequence similarity, the NFAT TADs are important structures that mediate the interaction between NFAT and partner proteins and are therefore very important for NFAT-mediated gene regulation. In this study, we observed that a NFAT1 TAD-C peptide (NFAT1 691-927) was unable to prevent CA-NFAT1-induced apoptosis by acting as dominant negative (Figure 4), suggesting that CA-NFAT1 may not interact with an NFAT partner protein through its TAD-C to induce apoptosis.

The NFAT1 TAD-C appears to be particularly important for the regulation of some apoptotic genes. Nur77, an activator of the mitochondrial apoptosis pathway, has been shown to induce apoptosis in immature timocytes and several types of cancer cells [33], [34], [35]. Interestingly, Nur77 is able to convert Bcl-2, a well-known anti-apoptotic protein that prevents the release of apoptogenic factors from mitochondria, into a pro-apoptotic protein [36]. The interaction of NFAT1 and the myocyte enhancer factor-2 (MEF2) through the NFAT1 TAD-C is required for activation of the Nur77 promoter [11]. Interestingly, this regulation was shown to be independent of NFAT1 binding to DNA [11]. Here, we assessed whether a mutant CA-NFAT1 that is unable to bind to DNA could induce apoptosis. Our analysis showed that CA-NFAT1 MutDBD did not induce apoptosis in NIH3T3 cells (Figure 5). This result demonstrated that binding to DNA and the transcriptional activity of CA-NFAT1 are required for induction of apoptosis in NIH3T3 cells.

NFAT1 is expressed in peripheral T cells, where it is involved in terminating the immune response by AICD. AICD is a form of apoptosis that is triggered by the activation of the extrinsic pathway by a death ligand. The NFAT1 TAD-C is important for the activation of one of these death ligands, the tumor necrosis factor-α (TNF-α) gene. NFAT1 binds to the κ3 promoter element of the TNF-α gene and activates TNF-α transcription [9]. The induction of the TNFα promoter by NFAT1 has been shown to be dependent on its TAD-C, as NFAT1 is able to transactivate the TNF-α promoter, while NFAT2 is not [27]. Interestingly, NFAT2 is only able to induce the TNF-α promoter when fused to the NFAT1 TAD-C [27]. The role of the NFAT1 TAD-C in the regulation of TNF-α has not been determined. Regulation of TNF-α by NFAT1 may explain the phenotype observed in CA-NFAT1-expressing and CA-NFAT2-TAD-C-NFAT1-expressing cells (Figs. 1, 2 and 6). As TNF-α is most commonly secreted, we assessed whether conditioned media from CA-NFAT1-expressing NIH3T3 cells induced apoptosis in wild-type NIH3T3 cells. Preliminary results from our group showed that supernatants from CA-NFAT1-expressing cells did not induce apoptosis in wild-type NIH3T3 cells (data not published). However, further studies are required to determine whether TNF-α is implicated in CA-NFAT1-mediated apoptosis in NIH3T3 cells.

AICD can also be activated by TRAIL and FasL. The TRAIL promoter possesses several putative NFAT binding sites [37]. In addition, TRAIL expression in stimulated T cells is sensitive to cyclosporine, a well-known inhibitor of calcineurin, suggesting a role for NFAT in its regulation [38]. However, no studies have reported direct regulation of TRAIL by NFAT1. FasL belongs to the tumor necrosis factor family and plays an important role in many immunological processes [39]. NFAT1 protein from nuclear extracts of activated T cells has been shown to bind two sites within the FasL promoter [10], and ectopic NFAT1 expression activates the FasL promoter [40]. However, there is no evidence of a role for the NFAT1 TAD-C in the regulation of the FasL promoter.

Although the TADs harbor the main differences of the NFAT members, the contributions of the TADs to NFAT-induced phenotypes are not well defined. Here, we showed that the NFAT1 TAD-C includes at least two NFAT death domains that mediate the pro-apoptotic function of NFAT1 and can also confer this function to another NFAT member. Apoptosis is implicated in several pathological conditions, including cancer. The evasion of mechanism of apoptosis is considered one of the cancer hallmarks [41]. Over the past two decades, several studies have demonstrated the roles of NFAT transcription factors in the regulation of cancer and have revealed that the functions of the NFAT members are not redundant [42]. NFAT1 has been reported to be a tumor suppressor gene, whereas NFAT2 has been reported to be an oncogene [20], [21], [43]. Thus, it appears that NFAT1 and NFAT2 have opposing effects. In our study, we showed that the opposing effects are mediated partially by a specific region of the NFAT1 TAD-C located between amino acids 699 to 850. The tumor suppressor activity of NFAT1 is dependent on its TAD-C [20] and may be conferred by NFAT1 TAD-C to another NFAT member, including a NFAT members described as a oncogene, such as NFAT2. Further studies will be necessary to determine the mechanisms by which NFAT1 induces apoptosis and which genes are differentially regulated by NFAT proteins in this context.

## Supporting Information

Figure S1 **Schematic representation of CA-NFAT proteins.** Schematic representation of the primary structures of CA-NFAT1 and CA-NFAT2. The mutated residues are indicated in the Figure. The crosshatched bar represents TAD-N (N-terminal transactivation domain), the grey bar represents NHR (NFAT-homology region), the black bar represents the DBD (DNA-binding domain), the white bar represents the TAD-C (C-terminal transactivation domain) and the red bar represents the nuclear localization signal (NLS) from T-antigen of simian virus 40 (SV40). (A) CA-NFAT1 representation. (B) CA-NFAT2 representation.
(TIF)

Figure S2 **All of the CA-NFAT1 truncated proteins show similar expression levels and have the expected molecular weight.** The total lysate of 4×10^5^ transduced NIH3T3 cells was obtained for analysis of all CA-NFAT1 truncated protein expression levels and molecular weight by Western Blot using anti-NFAT1 antibody.
(TIF)

Figure S3 **Wild-type NFAT1 does not induce apoptosis in NIH3T3 upon stimulation.** NIH3T3 cells were transduced with empty vector or retrovirus expressing NFAT1 or NFAT1 Δ699-850 and plated. After plating, cells were left unstimulated (Unst) or stimulated with PMA (20 nM) plus ionomycin (2 µM). NIH3T3 cells were stained with propidium iodide and analyzed for cell death by flow cytometry, 48 hours after stimulation. The percentage of cells in sub-G0 is shown in the graph.
(TIF)
