# Supplementary figures and images for: NFAT1 C-Terminal Domains Are Necessary but Not Sufficient for Inducing Cell Death
Source: PLoS One. 2012 Oct 26;7(10):e47868. doi: 10.1371/journal.pone.0047868 (PMC3482241; doi:10.1371/journal.pone.0047868)

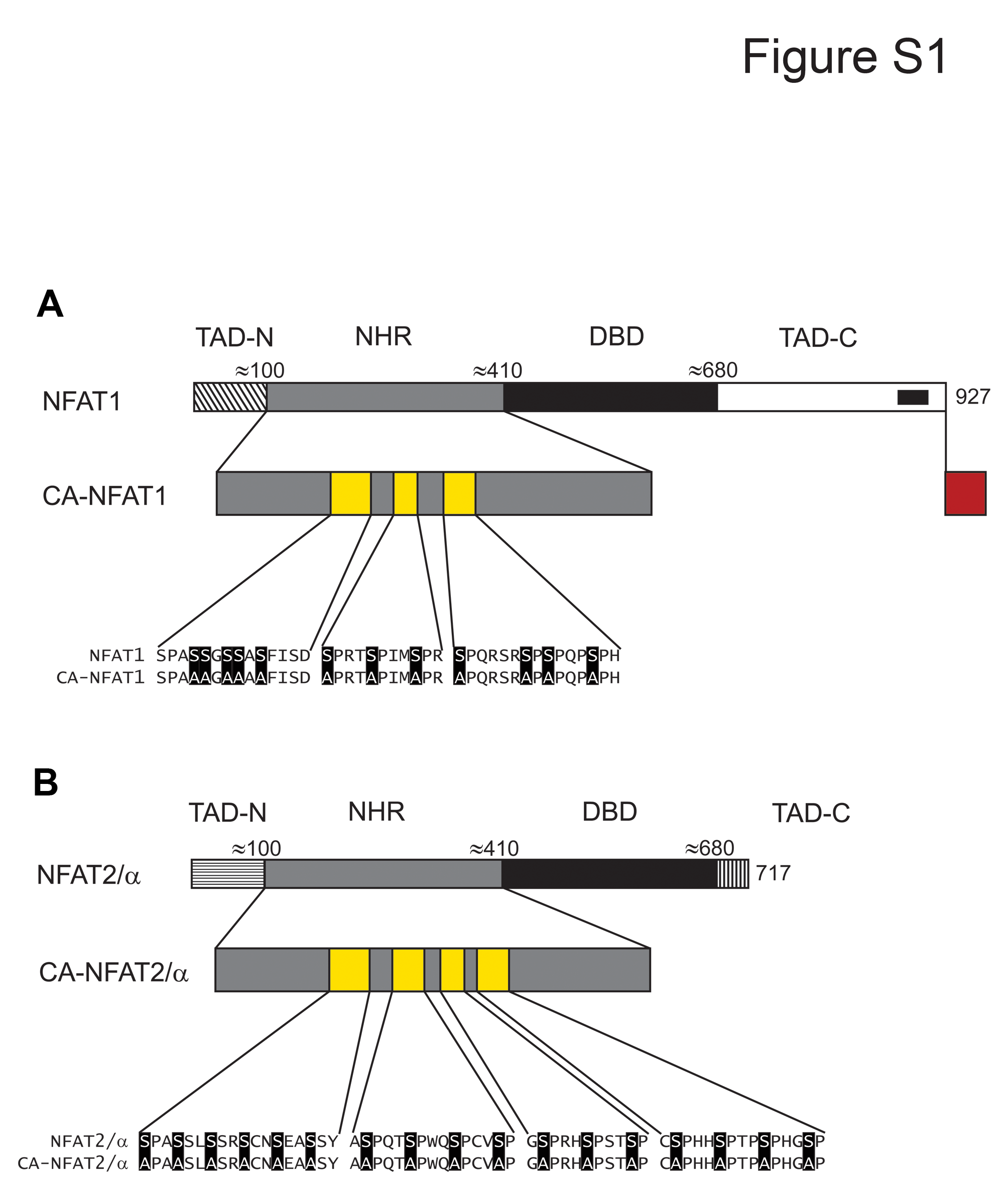

Supplement: Figure S1 — Schematic representation of CA-NFAT proteins. Schematic representation of the primary structures of CA-NFAT1 and CA-NFAT2. The mutated residues are indicated in the Figure. The crosshatched bar represents TAD-N (N-terminal transactivation domain), the grey bar represents NHR (NFAT-homology region), the black bar represents the DBD (DNA-binding domain), the white bar represents the TAD-C (C-terminal transactivation domain) and the red bar represents the nuclear localization signal (NLS) from T-antigen of simian virus 40 (SV40). (A) CA-NFAT1 representation. (B) CA-NFAT2 representation. (TIF) [file pone.0047868.s001.tif]

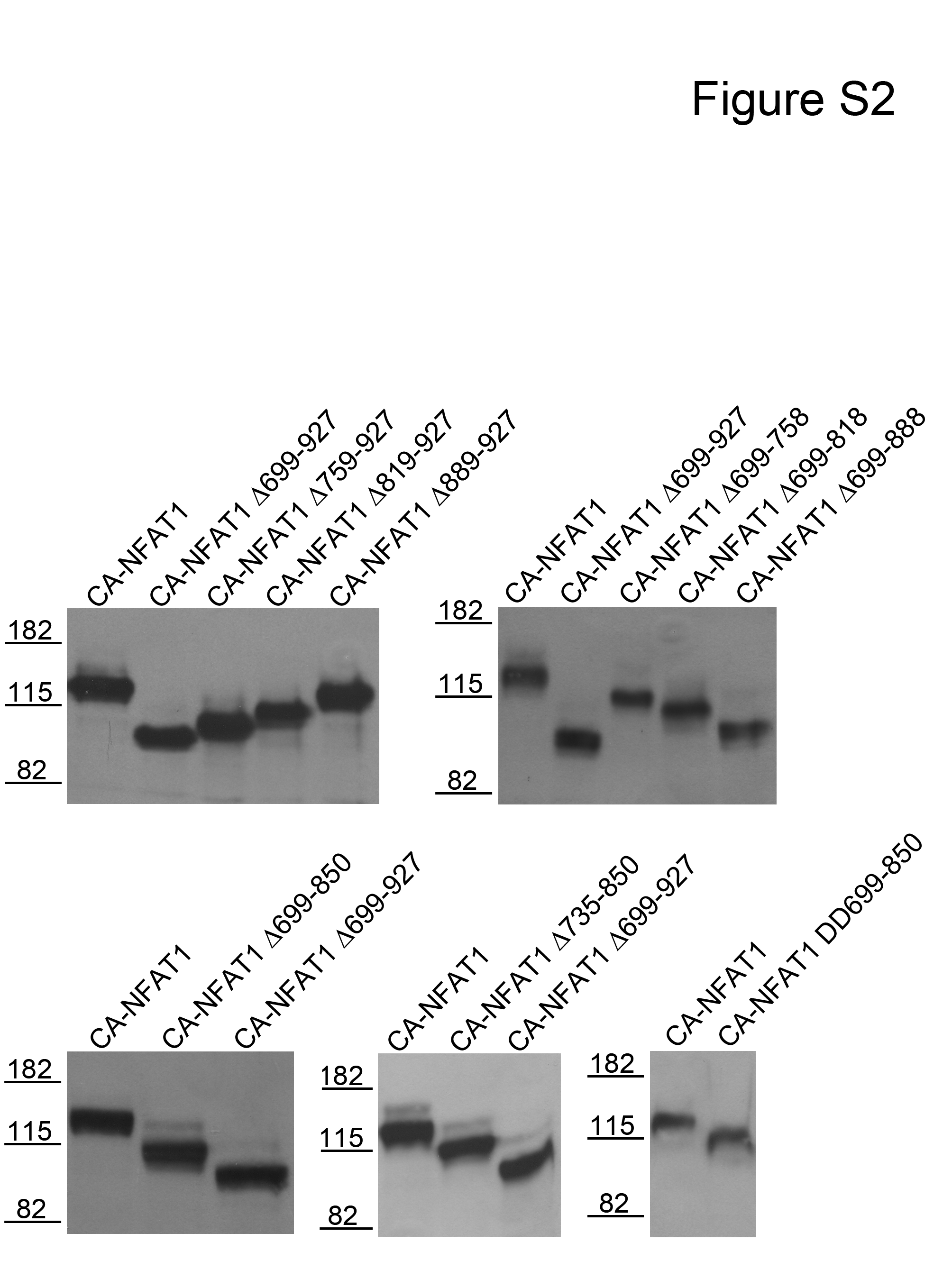

Supplement: Figure S2 — All of the CA-NFAT1 truncated proteins show similar expression levels and have the expected molecular weight. The total lysate of 4×10^5^ transduced NIH3T3 cells was obtained for analysis of all CA-NFAT1 truncated protein expression levels and molecular weight by Western Blot using anti-NFAT1 antibody. (TIF) [file pone.0047868.s002.tif]

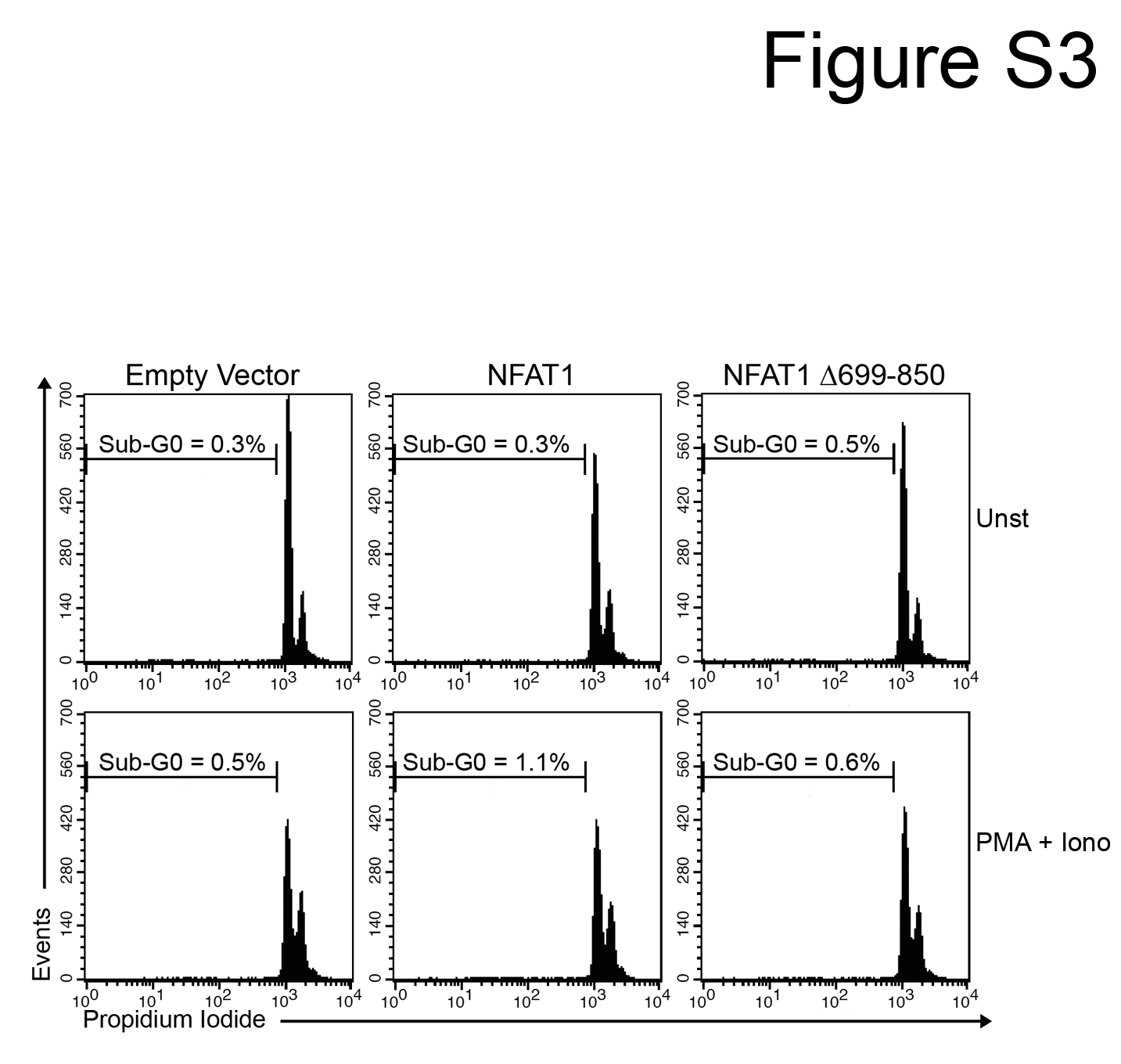

Supplement: Figure S3 — Wild-type NFAT1 does not induce apoptosis in NIH3T3 upon stimulation. NIH3T3 cells were transduced with empty vector or retrovirus expressing NFAT1 or NFAT1 Δ699-850 and plated. After plating, cells were left unstimulated (Unst) or stimulated with PMA (20 nM) plus ionomycin (2 µM). NIH3T3 cells were stained with propidium iodide and analyzed for cell death by flow cytometry, 48 hours after stimulation. The percentage of cells in sub-G0 is shown in the graph. (TIF) [file pone.0047868.s003.tif]
